# Regulation of extracellular vesicles for protein secretion in *Aspergillus nidulans*

**DOI:** 10.15698/mic2026.01.866

**Published:** 2026-01-28

**Authors:** Rebekkah E. Pope, Patrick Ballmann, Lisa Whitworth, Rolf A. Prade

**Affiliations:** 1 Department of Microbiology & Molecular Genetics, Oklahoma State University, Stillwater, OK 74078, USA; 2 Prüf-und Forschungsinstitut Pirmasens e.V., Marie-Curie-Strasse 19, 66953 Pirmasens, Germany; 3 OSU Microscopy Laboratory, Oklahoma State University, Stillwater, OK 74074, USA

**Keywords:** *Aspergillus nidulans*, extracellular vesicles, protein secretion, carbon source regulation, vesicle-mediated protein secretion, ER signal peptide, secretomes, xylanase, biofilm

## Abstract

Fungi were among the first eukaryotes to transition from aquatic to terrestrial life, developing multicellular hyphae, polar growth, and expanded secretomes for nutrient processing, defense, and symbiosis. We present a reliable method for purifying and characterizing extracellular vesicles (EVs) from *Aspergillus nidulans* and demonstrate that the induction of xylanase C is associated with increased EV release and EV-associated enzymatic activity. Using a mCherry reporter replacing xylanase C, we generalized this effect, showing that reporter induction increases EV production and reporter loading into EVs. This phenomenon primarily depends on the signal peptide (SP), suggesting that the induction of endoplasmic reticulum (ER)- trafficked proteins has a pronounced effect on EV production and cargo loading. We speculate that EV biogenesis may originate at the ER, where ER-translated proteins could be selectively loaded into vesicles and subsequently trafficked directly to the plasma membrane or through multivesicular bodies (MVBs). EV secretion is minimal in the first 24–48 hours but increases later in growth, coinciding with biofilm formation. This timing allows *A. nidulans* to modify the secretome, adapting it to new nutrient sources.

## INTRODUCTION

Fungi were among the earliest eukaryotes to transition from aquatic to terrestrial environments. This evolutionary shift was characterized by the loss of the flagellum and the emergence of a multicellular mycelial lifestyle, in which hyphae disseminated by polar growth colonize terrestrial ecosystems 1. During this transition, fungi also evolved to export a substantial portion of their metabolic portfolio to the extracellular space, developing secretomes specialized in complex-nutrient acquisition 2–4, self-protection from predators 5, 6, and development of symbiotic and parasitic relationships on terrestrial ecosystems 7.

Secretomes comprise all proteins exported beyond the plasma membrane, including those in the extracellular medium, within biofilms, and attached to the cell wall 8, 9. Mass spectrometry analysis of secretomes from *Aspergillus nidulans*, *Aspergillus niger,* and *Aspergillus fumigatus* on various carbon sources revealed unique extracellular protein counts ranging from 221 to 294 proteins 10–12. Secretome composition is a regulated and dynamic process that changes in response to environmental conditions (e.g., nutrients, pH, and oxidative stress) and other metabolic and environmental signals 2, 13, 14.

Endocytosis (membrane internalization) and exocytosis (vesicle-mediated secretion) regulate many of the cell interactions with the environment. Exocytosis involves the diffusion of intracellular vesicles to the plasma membrane, where their contents are released into the extracellular space 15. In fungi such as *Cryptococcus neoformans*, *Histoplasma capsulatum,* and *Candida albicans*, substantial evidence indicates that exocytosis is the main route for trans-cell wall macromolecule transport via multivesicular bodies (MVBs), which fuse with the plasma membrane and release cargo through conventional secretory pathways 16–18. Additionally, fungi release plasma membrane-derived blebs via outward budding of the plasma membrane, the microvesicle pathway, thereby generating extracellular vesicles 19–21.

Extracellular vesicles (EVs) have been recognized across various biological kingdoms for decades, though fungal EVs received little attention until recent years 21. Observed initially in *C. neoformans* and *A. nidulans* in the 1970s, EVs were often dismissed as cellular debris or artifacts 22–24. However, recent studies identify EVs as a critical mechanism for exporting diverse cargo across the plasma membrane 25. In fungi, EVs export proteins that contribute to the secretome, along with cell wall glycan components, pigments 23, nucleic acids such as miRNAs 26, and other signaling molecules 27.

Extracellular protein secretion initiates at the protein translation level in the cytoplasm via the recognition of an N-terminal signal peptide, which directs the nascent peptide to the endoplasmic reticulum (ER) lumen 28, 29. ER-translated proteins are packaged into intracellular vesicles (IVs), which bud off the ER membrane and travel towards various endomembrane compartments 30–33. Similarly, live-cell imaging in *A. nidulans* revealed that autophagosomes originate from ER-derived membranes 34.

In fungi, IVs are transported along microtubules from the ER to the Golgi apparatus, where secretory vesicles (SVs) are formed. These SVs travel to the Spitzenkörper at the hyphal tip, where they accumulate and fuse with the plasma membrane, releasing their content into the cell wall space 33, 35–41. SVs concentrated at the hyphal tip promote fungal growth by delivering phospholipids for membrane expansion, sugars, and cell wall assembly materials 42. In *Ustilago maydis,* exocytic vesicles were found that contain myosin-chitin synthase (Mcs1) as well as class VII chitin synthase and 1,3- β -glucan synthase 43, all of which are enzymes needed to build a cell wall from the outside in.

Exocytosis of molecules and proteins involved in polar growth and cell wall construction predominantly occurs at the hyphal tip, contributing to hyphal extension. However, the secretion of hundreds of metabolically active enzymes, which comprise the bulk of secretomes, remains unexplored. Notably, accumulating evidence suggests that exocytosis may also occur at alternative subapical cellular sites, such as septa 44, or involve a sub-population of vesicles that emerge from the ER membrane and are directed to the plasma membrane, bypassing the Golgi apparatus 45–48.

This study investigates how *A. nidulans* secretes metabolically essential proteins beyond the plasma membrane and structures its extracellular metabolic network. We observe that *A. nidulans* consistently releases high concentrations of EVs into the medium, regardless of the carbon source. Our findings reveal that *A. nidulans* continuously secretes EVs. However, the specific cargo loading of these EVs increases in parallel with the transcriptional activation of genes and secretion of ER-translated products, facilitating extracellular secretome adaptations in response to environmental changes. We present evidence that EV biogenesis originates at the ER, where they are loaded with newly translated proteins and travel directly to the plasma membrane for secretion. Moreover, we show that EV secretion increases at later growth stages and peaks at the onset of the stationary phase when nutrient adaptation becomes critical. Overall, our results demonstrate a strong link between the regulation of EV production and protein secretion in *A. nidulans*.

## RESULTS

### *A. nidulans* produces extracellular vesicles in two distinct size ranges, and their secretion is affected by the carbon source

**Figure 1:**
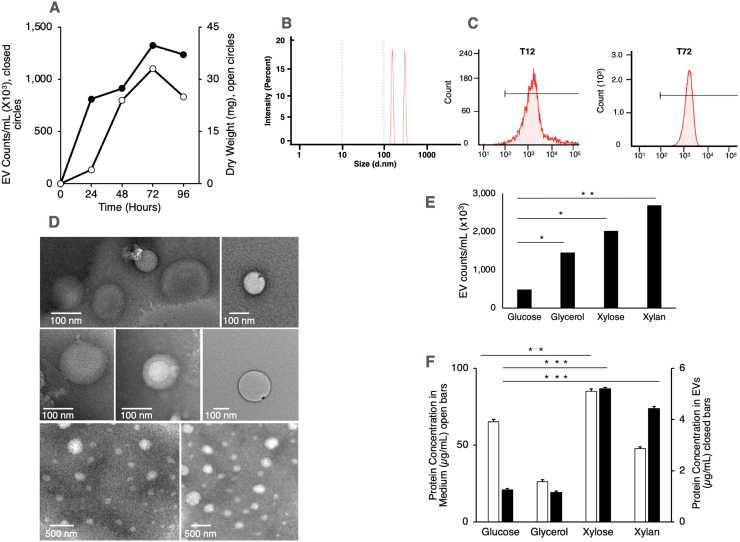
Characterization, quantification, and visualization of extracellular vesicles (EVs) released by *A. nidulans*. ** (A) ** Mycelial growth and EV secretion over time. *A. nidulans* conidia were cultured in liquid minimal medium with 1% glucose at 37 ${}^{\circ }$ C under stationary conditions. Biomass accumulation was measured as dry weight (open symbols). EVs were purified from cell-free supernatant (12,000 ×g) by ultrafiltration (300 kDa MWCO), labeled with CellBrite-488, and quantified by FFC (closed symbols). EV secretion peaked at 72 hours. **(B)** Dynamic light scattering (DLS) analysis of purified EVs (from **1A**) showing two predominant size ranges: 100–200 nanometers and 300–400 nanometers. **(C)** Fluorescence flow cytometry (FFC) histograms of purified EVs (from **1A**) labeled with CellBrite-488 after 12 hours (T12) and 72 hours (T72) of mycelial growth. EVs were detectable as early as 12 hours, with clearer resolution at 72 hours (see Supplemental Figure S1). **(D)** TEM imaging of purified EVs. Purified EVs were fixed for transmission electron microscopy (TEM) from *A. nidulans* grown in xylose medium for 48 hours. EVs appeared in two size groups, ∼100 nm and ∼200 nm. **(E)** EV secretion in different carbon sources. Mycelia were grown for 48 hours in glucose, glycerol, xylose, or xylan, and purified EVs labeled with CellBrite-488 and quantified by FFC (EVs/mL). **(F)** Estimation of total secreted protein and EV-associated proteins. Mycelia were grown for 48 hours in glucose, glycerol, xylose, or xylan, and the protein content was measured in purified EVs (closed bars) and in the intact medium (open bars).

An optimized cultivation system is essential for isolating EVs from the culture medium. *A. nidulans* conidia were inoculated in Petri dishes containing a liquid minimal medium with 1% glucose as the carbon source and incubated at 37 ${}^{\circ }$ C under stationary (non-shaking) conditions. This approach minimizes mechanical stress, reduces cellular damage and debris, and enables the development of a floating mycelial mat, thereby limiting the formation of submerged hyphae and pellets. Medium samples from liquid cultures were centrifuged at 12,000 × g to obtain a cell-free supernatant (CFS). The CFS was ultrafiltered (300 kDa MWCO) to separate EVs from soluble proteins, larger molecular complexes, and aggregates. It was then washed with PBS (pH 7.4) and stored at 4 ${}^{\circ }$ C for subsequent analysis. Figure 1**A** shows mycelial growth and EV secretion over time in our EV-optimized cultivation system.

In purified EV fractions, we detected only two distinct particle sizes by dynamic light scattering (DLS), 100–200 nm and 300–400 nm (Figure 1**B**). No additional peaks were detected in lower light-scattering regions, confirming the absence of protein aggregates, larger complexes, ribosomes, and cellular debris. Supplemental Figure S1 presents the DLS spectra of unpurified and partially purified samples, highlighting impurity peaks alongside the characteristic particles in the 100–200 and 300–400 nanometer ranges.

Fluorescence Flow cytometry (FFC) analysis (Figure 1**C**) of purified EV preparations labeled with CellBrite-488 revealed a single fluorescence peak in samples from cultures grown for 12 and 72 hours. The 12-hour samples showed more noise and background, while the 72-hour samples exhibited a more uniform fluorescence distribution, corresponding to a higher EV count. Because EVs are too small to be counted individually, we used FFC to quantify CellBrite-488-labeled EVs, which are reported throughout this study as EV counts/mL.

Our DLS and FFC analyses suggested the production of uniformly sized EVs. We directly visualized purified *A. nidulans* EV preparations using transmission electron microscopy (TEM) to confirm this assumption. Figure 1**D** displays purified EVs from the culture medium, with sizes matching the EV values obtained using DLS (100–200 nm).

In *A. nidulans* grown vegetatively on glucose, EV secretion begins at 24 hours and peaks at 72 hours, reaching 1.33 × 10 ${}^{6}$ EV counts/mL (Figure 1**A**). However, when *A. nidulans* was grown on different carbon sources (Figure 1**E**), EV accumulation at 48 hours (mid-exponential phase) was approximately 0.473 × 10 ${}^{6}$ , 1.4 × 10 ${}^{6}$ , 2.0 × 10 ${}^{6}$ , and 2.7 × 10 ${}^{6}$ EV counts/mL for glucose, glycerol, xylose, and xylan, respectively. Figure 1**F** depicts the distribution of total secreted proteins in the medium (open bars) versus those encapsulated in EVs (closed bars). During mid-exponential growth (48 hours) on glucose, 1.94% of secreted proteins were EV-associated, compared to 4.41%, 6.11% and 9.28% for glycerol, xylose, and xylan, respectively.

To investigate EV cargoes, *A. nidulans* (XynMC11) was grown in xylose for 72 hours, and EVs were purified from 250 milliliters of culture medium. Proteins in the EV fraction were analyzed by LC-MS/MS-based proteomics (see Methods for details). We anticipated that most identified proteins would represent genuine EV cargoes, alongside a smaller subset of extracellular enzymes, some with or without signal peptides. Given that EVs were purified from XynMC11 (see result section “Construction of the EV-cargo reporter strain XynMC11 and derivatives”) grown on xylose, we also expected detection of the EV-reporter cargo protein mCherry.

Table 1 summarizes the proteins identified in EV preparations from strain XynMC11. A total of 237 unique peptides corresponding to 46 unique proteins were detected, of which 30 (65%) encode signal peptides (in the genome), and 16 (35%) do not. As expected, the EV cargo protein mCherry was identified. A complete list of proteins detected by LC-MS/MS, along with the raw data, is provided in Supplemental Table S1.

These results indicate that EV purification using stationary mycelial cultures and 300 kDa cutoff ultrafiltration effectively enriches EV-associated proteins. To account for unavoidable contaminants, we used complementary approaches: EVs were labeled with CellBrite-488 membrane stain and quantified by FFC, while fluorescence spectrophotometry measured cargo abundance in liquid cultures.

**Table 1:** Summary of LC-MS/MS proteomics on purified EVs.

**GO**	unique proteins		unique peptides	
		
	count	%	count	%
proteins/peptides detected	46	-	237	-
**with Signal Peptide**	**30**	**65%**	**152**	**64%**
EV cargo reporter (mCherry)	1	2%	9	4%
enzyme	21	46%	117	49%
uncharacterized	4	9%	14	6%
cell wall/biofilm formation	4	9%	12	5%
**no Signal Peptide**	**16**	**35%**	**85**	**36%**
chaperone HSP90	1	2%	6	3%
cell wall remodeling	3	7%	30	13%
stress response	4	9%	22	9%
enzyme	3	7%	11	5%
uncharacterized	5	11%	16	7%

**GO**: Biochemical function/process extracted from GO Terms. Supplemental **Table S1** displays LC-MS/MS raw collected proteomics data and a curated list of peptides and proteins detected.

### Carbon source regulates EV cargo loading

The observation that EVs respond to the carbon source in both overall quantity (Figure 1**E**) and proportion of encapsulated protein cargo (Figure 1**F**) suggests that the availability of the carbon source may regulate the secretion system(s) responsible for EV cargo loading. To test this hypothesis, we focused on hemicellulose (xylan) as an adaptive carbon source for fungi. Utilization of xylan by fungi requires recognition of the substrate, induction of xylanase encoding genes (e.g., *xlnA*, *xlnB*, *xlnC,* and *xlnD*), translation, and secretion of xylanases to degrade xylan into xylose, the primary accessible xylan-derived carbon source 49, 50. Xylose is both the inducer for xylanase production and a growth-supporting carbon source 51, 52.

We cultured two strains, A773 (wild-type reference) and PFI-XLN7, an engineered strain for hemicellulose adaptation experiments. PFI-XLN7 constitutively expresses the XlnR regulator, thus causing xylanase (*xlnC*) overproduction in response to inducers like xylose or xylan 51. Both strains were grown in a minimal medium with 1% glucose, 1% xylose, or 1% xylan for 72 hours. We measured total xylanase activity in the medium and in purified extracellular vesicles (EVs) and quantified secreted EVs.

**Figure 2:**
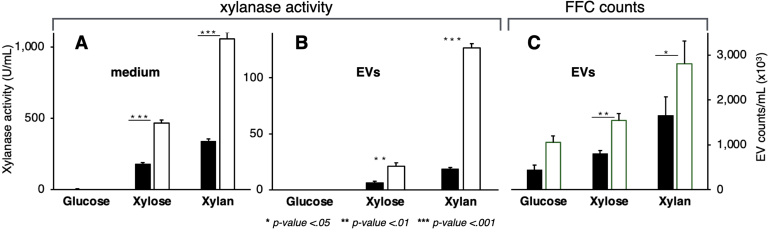
The carbon source regulates the loading of EV cargo. Two strains of *A. nidulans* were used to detect total xylanase activity present in the crude extracellular medium or contained in EVs: A773 (wildtype-like reference strain—closed bars) and PFI-XLN7 (open bars), which constitutively expresses the XlnR regulator to drive xylanase (*xlnC*) overproduction in response to inducers like xylose or xylan 51. Liquid medium samples were collected from A773 and PFI-XLN7 grown in glucose, xylose, or xylan for 72 hours, and total xylanase activity was determined in the crude harvested medium (**A**) and purified EVs (**B**) along with FFC counts of EVs labeled with CellBrite-488 (**C**).

In A773, xylanase activity in the medium was negligible in glucose-grown mycelia (7 ± 10 U/mL) but increased to 180 ± 9 U/mL in xylose and 338 ± 16 U/mL in xylan, representing 27- and 51-fold induction, respectively (Figure 2**A**, closed bars). EV-associated total xylanase activity was similarly low in glucose (0.3 ± 0.4 U/mL) but increased to 6 ± 1 U/mL in xylose and 19 ± 1 U/mL in xylan, corresponding to 23- and 68-fold induction, respectively (Figure 2**B**, closed bars). In PFI-XLN7, a similar trend was observed, with even higher induction levels, consistent with the strain’s xylanase overproduction. Xylanase activity in the medium was enhanced 124-fold in xylose and 279-fold in xylan (Figure 2**A**, open bars), while EV-associated xylanase activity showed 15- and 88-fold increases, respectively (Figure 2**B**, open bars). When switching the carbon source from glucose to xylose or xylan, total EV counts increased significantly in both strains, A773 and PFI-XLN7 (Figure 2**C**). These results support the idea that transcriptional induction and post-translational mechanisms actively load EVs with newly translated enzymes from the ER. Moreover, we observed a correlation between enzyme induction and EV secretion.

To acquire more evidence that EV cargo loading is a consequence of transcriptional induction and is linked to the post-translational secretion mechanism, we constructed a strain that replaced the *xlnC* mature protein coding sequence (AN1818) with the *mCherry* coding sequence. The recombinant strain, XynMC11, translates mCherry instead of xylanase C (see **Figure S2**) and retains all the native genetic features of the native AN1818 locus, including the promoter, 5’- and 3’- UTRs (untranslated regions on the mRNA), as well as the original *xlnC* signal peptide, which directs the translation of mCherry to the ER. By keeping the mRNA features intact as much as possible, with the only difference being the replacement of the xylanase C coding sequence with mCherry, we aimed to ensure that the protein would be directed to the ER and secreted into the extracellular space in the same manner as xylanase C.

**Figure 3:**
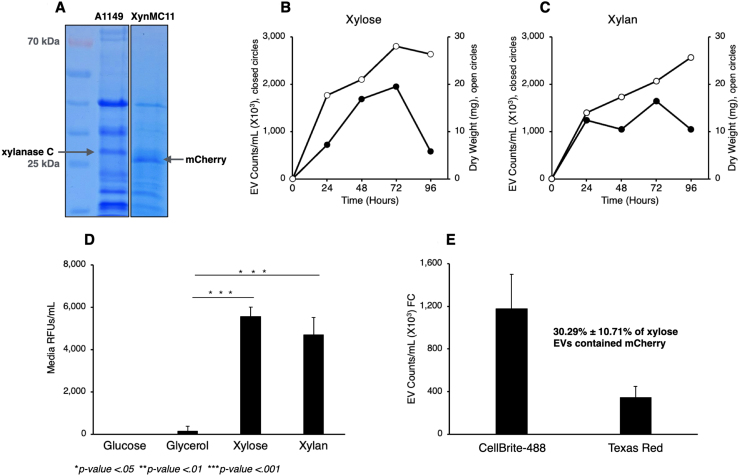
Characterization of the XynMC11 strain expressing *mCherry* under the native *xlnC* promoter. The *xlnC* coding region in the wildtype strain A1149 was replaced with *mCherry*, generating the XynMC11 strain, which expresses and secretes mCherry under native *xlnC* regulatory elements, including the original signal peptide. **(A)** SDS-PAGE of total secreted proteins (secretome) after 72 hours in xylose medium. A1149 showed a 34 kDa xylanase C band, while XynMC11 displayed a 27 kDa mCherry band. **(B, C**) Growth (dry weight, open circles) and EV secretion (CellBrite488-labeled, closed circles) of XynMC11 in xylose (**B**) and xylan (**C**). **(D)** Carbon source dependence of mCherry secretion. After 48-hour growth, mCherry fluorescence (RFU; excitation: 587 nm, emission: 610 nm) was high in xylose and xylan (inducing conditions) but minimal in glucose and glycerol (repressing conditions). **(E)** Quantification of total EVs (CellBrite 488) and mCherry-positive EVs (Texas Red).

Indeed, our results with XynMC11 indicated that mCherry was regulated, expressed, and secreted identically to xylanase C. Figure 3**A** shows the total extracellular protein profile of A1149 (wildtype reference strain – A1149 is the parent strain of XynMC11) and XynMC11, which indicates that XynMC11 no longer secretes xylanase C, but mCherry instead.

Figures 3**B**–**C** show the temporal growth kinetics, dry weight, and CellBrite-488-labeled EV counts of XynMC11 grown in the presence of xylose (**B**) and xylan (**C**). In both alternative carbon sources, dry weight did not change significantly, and EV secretion rates were comparable.

Figure 3**D** shows the amount of mCherry secreted into the medium, detected as mCherry RFU units (ex. 587 nm, em. 610 nm) in media containing 1% glucose, glycerol (a weaker carbon catabolite repressor), xylose, or xylan. The xylose and xylan media had significantly higher RFU values—39-fold and 33-fold, respectively—than glycerol. As expected, no significant mCherry fluorescence was detected in the glucose media, as it is a repressive carbon source.

Figure 3**D** quantifies the total secreted EVs (CellBrite-488 labeled) and EVs containing mCherry (quantified using Texas Red detection). While 1,178 × 10 ${}^{3}$ EVs were counted with CellBrite-488, at least 343 × 10 ${}^{3}$ were counted with mCherry fluorescence, suggesting that 30 ± 10% of EVs carried mCherry as cargo protein. Our findings are supported by other EV research indicating regulated routes and mechanisms for EV biogenesis, which depend on the cargo’s physiological role 53, 54.

We then expanded our analysis beyond XynMC11, which expresses mCherry at wildtype levels (see Figure 3), to include the strain XlnR7MC7. This strain is similar to XynCM11 except that it overproduces mCherry due to constitutive expression of the XlnR regulator analogous to the PFI-XLN7 strain (for construction of XlnR7MC7, see Methods). Liquid medium samples were collected from cultures of XynMC11 and XlnR7MC7 grown in glucose or xylose for 48 hours. We then quantified mCherry fluorescence in the medium and within isolates (EVs). Total EVs were counted via FFC.

Table 2 shows that XynMC11 (wildtype-like) mCherry release into the medium increased 15-fold on xylose, and mCherry trapped in EVs increased 34-fold, while in XlnR7MC11 (overexpression strain), mCherry in the medium increased 36-fold and mCherry in EVs increased 131-fold, respectively. The induction levels of the engineered strain at least doubled in mCherry in the medium; mCherry trapped in EVs almost quadrupled (Table 2). However, the fraction of EVs containing mCherry remained unchanged at 41% and 44%, respectively. These results suggest a strong link between mCherry (reporter) transcriptional regulation and post-transcriptional processing, which culminates in the loading of EVs with mCherry and their secretion into the extracellular space.

**Table 2:** The carbon source regulates mCherry secretion and the EV cargo loading mechanism.

Carbon source	Strain	mCherry (RFUs/mL)		% in EVs	EVs
				
		medium	EVs		counts/mL
glucose	XynMC11	66.3 ± 18.77	11.7 ± 11.06		1.34 x 10 ${}^{4}$
xylose		974.0 ± 7.00	402.0 ± 80.98	41	6.57 x 10 ${}^{3}$
**xylose fold induction**		**15**	**34**		
glucose	XlnR7MC7	46.7 ± 4.93	5.7 ± 9.81		2.72 x 10 ${}^{4}$
xylose		1,695.7 ± 27.46	742.3 ± 115.50	44	2.80 x 10 ${}^{4}$
**xylose fold induction**		**36**	**131**		

### mCherry-loaded EV detection on micrographs following growth in xylose

**Figure 4:**
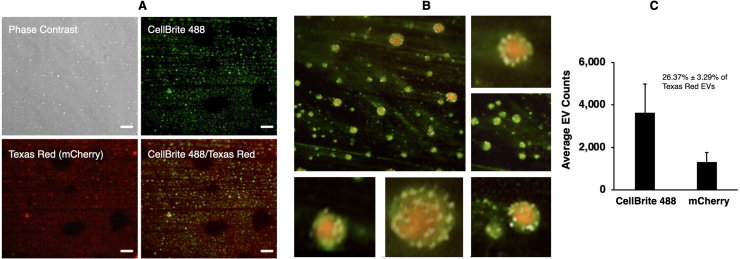
EVs detected with mCherry on micrographs agree with mCherry-containing EVs detected with FFC. Purified EVs from XynMC11 cultures grown in xylose medium were labeled with CellBrite-488, mounted on microscope slides, and visualized by fluorescence microscopy using CellBrite (membrane) and Texas Red (mCherry) channels. **(A)** Fluorescence microscopy of isolated XynMC11 EVs shows in Phase Contrast, CellBrite, Texas Red, and merged (CellBrite/Texas Red). **(B)** High magnification merged images (CellBrite/TexasRed) of representative XynMC11 EVs. **(C)** Quantitative analysis of CellBrite (all EVs) and Texas Red (only mCherry carrying EVs) signals in micrographs (**A**) showed that ∼26% of all EVs contain mCherry cargo, indicating selective packaging of transcriptionally regulated protein into EVs.

Our FFC results suggested that a subset of EVs was loaded with mCherry. Hence, we directly observed purified EVs using fluorescence microscopy. We imaged EV smears under phase-contrast, GFP (CellBrite 488), and Texas Red (mCherry) illumination (Figure 4**A**). CellBrite 488 stained 100 nm and 200 nm EVs, while mCherry was detected within fewer EVs, mainly those closer to 200 nm in size. Figure 4**B** shows various magnifications of a GFP/Texas Red (CellBrite 488/mCherry) overlay image, highlighting structured membrane staining (CellBrite 488) and mCherry inside EVs as cargo. Figure 4**C** displays a statistical analysis of EVs stained with CellBrite 488 and those illuminated with a Texas Red filter (mCherry) and stained with CellBrite 488. Approximately 26 ± 3% of the EV population contained mCherry, confirming earlier FFC quantitation with XynCMC11 (Figure 3**E**).

The data in Figure 4 show that specific cargos detected in EVs directly adapt in response to a change in carbon source, from glucose to xylose or xylan, representing ∼30% of the EV population. Our findings support the idea that EVs transport transcriptionally induced enzymes, such as xylanase or mCherry (where the XlnC enzyme is replaced by the mCherry reporter), out of the cell to help the fungus adapt to different carbon sources and produce metabolically accessible nutrients.

### Spatiotemporal endomembrane dynamics of mCherry localization and EV secretion during late vegetative growth

Upon induction, mycelia synthesize the corresponding adaptive enzyme (or mCherry reporter), which is packaged into EVs and secreted into the extracellular space (Figures 3–4 and Table 2). To determine the timing of EV release, approximately 100 XynMC11 conidia were inoculated onto xylose-supplemented agar pads and incubated at 37 ${}^{\circ }$ C for 120 hours. Fluorescence (Calcofluor and Texas Red filters) and phase contrast microscopy revealed a temporal pattern of mCherry localization consistent with growth kinetics in Figures 1 and 3.

**Figure 5:**
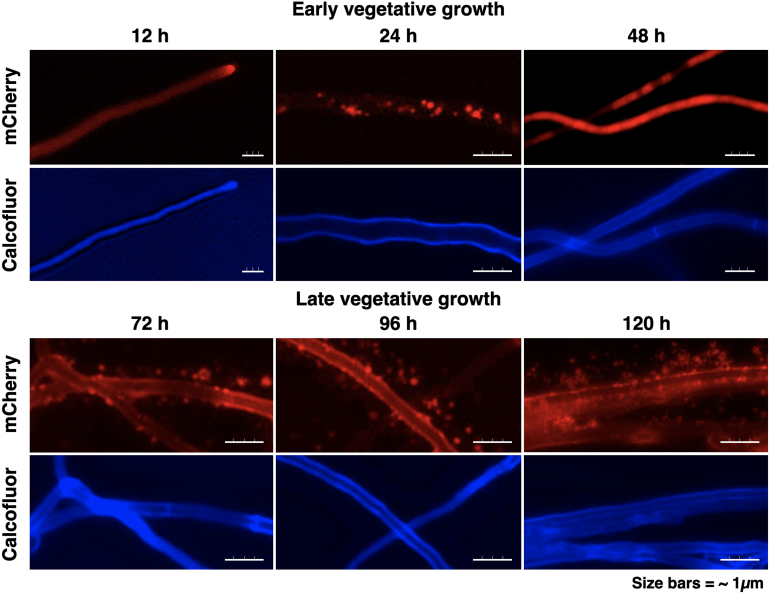
Temporal localization of mCherry during vegetative growth. During early vegetative growth, mCherry accumulates within intracellular endomembrane structures, whereas at later stages it is secreted into the extracellular space via EVs. XynMC11 was grown on xylose agar pads on microscope slides, and mCherry localization was analyzed by fluorescence microscopy. Single-channel images (mCherry and calcofluor) are shown separately, highlighting mCherry-labeled cargo and the calcofluor-stained hyphal cell boundaries. In young hyphae (12–48 hours), mCherry fluorescence marked intracellular endomembrane structures of various shapes and sizes near hyphal tips, with no extracellular signal. By 72 hours, large structures disappeared, and numerous smaller (<500 nm) mCherry-labeled vesicles appeared at the plasma membrane, cell wall, and throughout the extracellular space.

Figure 5 shows that at 12 hours, mCherry localized at the hyphal apex, by 24 to 48 hours, it accumulated in large (1–2 μ m) endomembrane structures throughout the hyphal cytoplasm with no extracellular signal. After 60 hours, abundant extracellular mCherry-labeled vesicles (∼500 nm) appeared at the plasma membrane, cell wall, and in the surrounding medium, indicating active EV secretion.

These findings suggest that early growth involves intracellular trafficking of mCherry-labeled EVs toward the hyphal tip. At the same time, late-stage accumulation at the membrane and in the extracellular space reflects a transition to enhanced EV-mediated secretion during hyphal maturation.

### Role of biofilm formation in EV-mediated enzyme retention and secretion

Yeast and filamentous fungi can form biofilms to aid nutrient acquisition, adherence, and protection against environmental stressors 55, 56. Figure 5 clearly shows that older hyphae (72 hours) release substantial amounts of EVs into the extracellular space. According to Figure 3, they shed approximately 2 × 10 ${}^{6}$ EVs/mL into the medium under xylose or xylan-adaptive conditions. It is unclear whether *A. nidulans* releases EVs directly into the environment, allowing them and their enzyme cargoes to disperse freely, or whether stress-induced biofilm formation traps EVs, thereby retaining enzyme activity near the cell.

To evaluate whether mCherry-loaded EVs are retained within biofilm structures or released into the extracellular environment, XynMC11 conidia were inoculated into a minimal liquid medium containing 1% xylose. The conidia suspension was then transferred onto coverslips at the bottom of a Petri dish, following a modified protocol adapted from Shay *et al.* 57.

**Figure 6:**
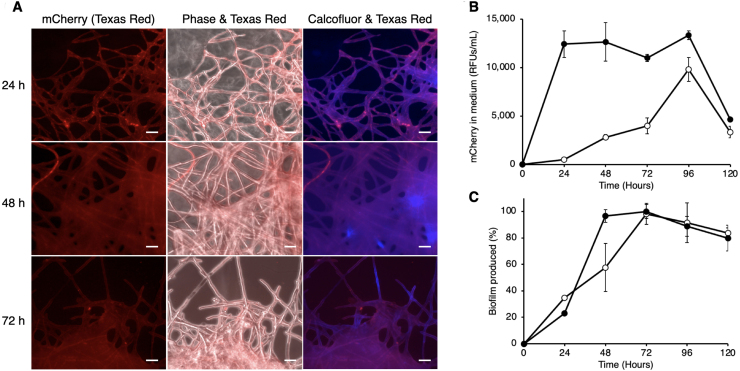
mCherry accumulation in biofilm during late vegetative growth. XynMC11 spores were inoculated onto coverslips placed at the bottom of Petri dishes containing 1% xylose. Following incubation, mycelia were stained with Calcofluor and mounted for fluorescence microscopy. **(A)** Fluorescence microscopy at 24, 48, and 72 hours revealed progressive biofilm development (Calcofluor) and increasing mCherry fluorescence (Texas Red). Phase/Texas Red overlays indicate that the mCherry is primarily localized to the biofilm matrix. **(B)** Relative fluorescent units (RFUs) of secreted mCherry were quantified in glucose (open circles) and xylose (closed circles) media every 24 hours for 120 hours. XynMC11 secreted higher mCherry levels in xylose than in glucose at all time points, with a peak at 48 hours coinciding with maximal biofilm formation. **(C)** Biofilm biomass determined by crystal violet assays in 6-well plates, increased up to 96 hours in both glucose (open circles) and xylose (closed circles) media. Biofilm levels were comparable between conditions, although xylose supported slightly greater biomass, consistent with the elevated EV counts observed at 48 hours (Figures 1**E** and 1**F**).

Figure 6**A** shows fluorescence microscopy of XynMC11 on coverslips in xylose media at 24, 48, and 72 hours. The Texas Red filter detects mCherry, and phase contrast defines cellular structure. Calcofluor White (using a DAPI filter) is a non-specific fluorochrome that binds to chitin and various β -linked polysaccharides present in biofilms 57.

The presence of mCherry within EVs (Figure 3 and 4) and the biofilm (Figure 6**A**) indicates that EVs deliver cargo to the biofilm, maintaining active enzymes near hyphal surfaces for nutrient uptake. However, detecting mCherry in the extracellular liquid (Figure 3**D** and Table 2) suggests that some enzymes are not confined to the biofilm but also diffuse freely in the liquid environment.

To investigate the correlation between biofilm production, mCherry, and EV secretion, XynMC11 was grown in 6-well plates using 1% glucose or xylose in liquid media. Media were analyzed for mCherry fluorescence every 24 hours for 120 hours (Figure 6**B**), and biofilm formation (Figure 6**C**) was quantified using crystal violet staining 58. mCherry peaked at 96 hours, with xylose showing 1.35-fold more mCherry than glucose, while early growth (24–48 hours) in xylose yielded significantly higher mCherry than glucose. Biofilm production and mCherry secretion trends peaked at 72 and 92 hours, respectively, suggesting *A. nidulans* may utilize EVs to deposit enzymes into biofilms on solid surfaces, potentially aiding nutrient uptake.

### Impact of signal peptide removal on EV production and mCherry localization

In eukaryotes, an N-terminal signal peptide (SP) directs polypeptides to the ER for translation in the secretion pathway 28, 29, 59. To assess the role of the SP in mCherry loading into EVs, the SP was removed from strain XynMC11, creating strain Xyn Δ SP, which is identical to XynMC11 but lacks the native *xynC* SP.

**Figure 7:**
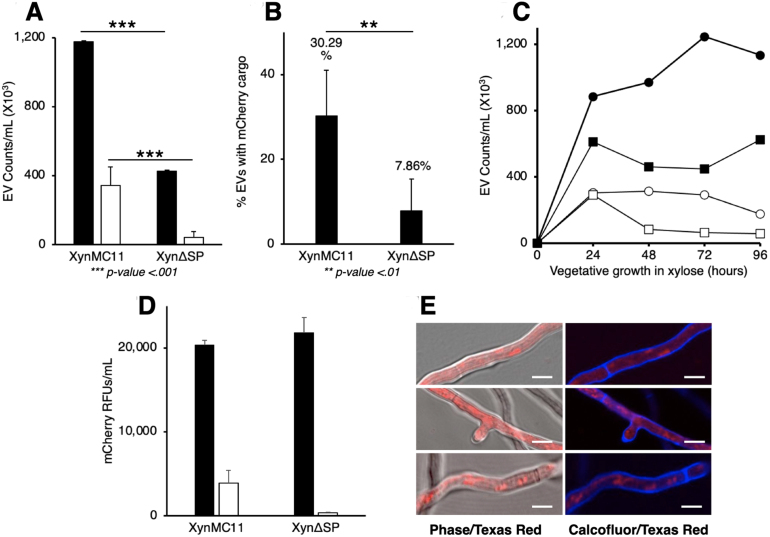
Removing the native *xlnC* signal peptide in XynMC11 results in fewer extracellular vesicles (EVs) being secreted and fewer EVs containing mCherry. XynMC11 was genetically modified to remove the native *xlnC* signal peptide (SP), resulting in Xyn Δ SP, which replaces the *xlnC* open reading frame (*ORF)* with *mCherry* lacking an SP while retaining the native untranslated region (UTR) and promoter. **(A)** XynMC11 and Xyn Δ SP were grown in a xylose liquid medium for 48 hours. EVs were isolated, labeled with CellBrite488, and analyzed by FFC to quantify total EVs (closed bars) and mCherry-containing EVs (open bars). Xyn Δ SP secreted fewer total extracellular vesicles (EVs) and mCherry-containing EVs compared to XynMC11. **(B)** FFC data showed Xyn Δ SP secreted a lower percentage of mCherry-containing EVs relative to total EVs than XynMC11. **(C)** Time course of EV secretion in two strains, XynMC11 with SP (circles) and Xyn Δ SP without SP (squares), was measured throughout the vegetative life cycle -up to 96 hrs. Closed symbols are for CellBrite-labelled EVs, and open symbols are for mCherry-containing EVs. **(D)** mCherry fluorescence (RFUs) was measured in an unfiltered medium (closed bars) and isolated EVs (open bars). Xyn Δ SP had fewer mCherry RFUs in EVs but showed levels similar to those in the medium compared to XynMC11. **(E)** Fluorescence microscopy of Xyn Δ SP grown on a xylose agar pad revealed mCherry localized primarily in the cytoplasm, unlike the extracellular localization seen in XynMC11 (Figure 5).

Figure 7**A** shows CellBrite 488-stained membranes (with FITC as the positive control and fluorescent channel) via FFC (closed bars), and mCherry detected with Texas Red (open bars) of XynMC11 and Xyn Δ SP grown for 72 hours in liquid xylose medium. The XynMC11 strain, which retained the native signal peptide, produced 2.75 times more EVs than Xyn Δ SP. Additionally, XynMC11 secreted 7.97 times more mCherry-containing EVs than Xyn Δ SP, which still produced mCherry-positive EVs but at significantly lower levels. The percentage of mCherry-containing EVs relative to the total EV count was also quantified (Figure 7**B**). Approximately 30% of XynMC11 EVs contained mCherry, compared to only 7.86% in Xyn Δ SP, a 3.85-fold difference (Figure 7**B**).

Figure 7**C** confirms the result shown in Figure 7**A** and Figure 7**B**, where the absolute amount of EVs (stained with CellBrite) and the relative amount of mCherry-loaded EVs (Texas Red) are significantly reduced in the absence of a signal peptide (Xyn Δ SP) throughout the vegetative growth cycle (up to 96 hours). Interestingly, this reduction in overall EV secretion in Xyn Δ SP is less accentuated in the first 24 hours, which coincides with the accumulation of mCherry in the apical region of the hypha (Figure 5).

Figure 7**D** shows that mCherry RFUs (closed bars) in xylose media were similar for XynMC11 and Xyn Δ SP after 72 hours of growth (no significant difference). However, mCherry RFUs in isolated EVs (open bars) from XynMC11 were 11.3 times greater than those from Xyn Δ SP. These results indicate that removal of the SP reduces EV production, suggesting that EVs carrying hydrolytic enzymes, such as xylanase C, originate from the ER membrane and contain freshly translated protein cargo destined for secretion.

To assess mCherry localization without a signal peptide (SP), Xyn Δ SP was grown on xylose agar pads and imaged (Figure 7**E**). Micrographs (Phase contrast/Texas Red (mCherry) filter overlay and DAPI (Calcofluor White)/mCherry overlay) showed mCherry primarily in the cytoplasm, unlike the intracellular spherical structures observed in XynMC11 (Figure 5). These results align with the lower mCherry fluorescence in EVs (Figure 7**D**), indicating that SP removal prevents mCherry from being incorporated into ER-derived EVs. The absence of an SP disrupts vesicle-mediated endomembrane pathways, leaving most mCherry free in the cytoplasm rather than encapsulated in vesicles.

## DISCUSSION

This study examines the role of vesicles in protein secretion in *A. nidulans* and their contribution to shaping the extracellular secretome in response to environmental changes. Our results describe how hyphae engage with EVs as the principal negotiators of extracellular protein secretion, highlighting adaptations in biogenesis, cargo selection, and nutrient acquisition.

Fungal EVs were first observed in the 1970s but were often dismissed as artifacts 22–24. We optimized their isolation using a stationary liquid culture that promotes a floating mycelial mat, reducing mechanical stress and debris. Figure 1**A** shows biomass (mycelial mat) accumulation and EV-secretion in a vegetative growth kinetics experiment using our EV-optimized cultivation system.

Purified EVs from these cultures can be detected by DLS, FFC, and TEM microscopy (Figures 1**B**, 1**C**, and 1**D**). FFC revealed abundant EVs, with secretion peaking at the stationary phase, and modulated by carbon source complexity (Figures 1**A**, 1**E**, and 1**F**), indicating a correlation between EV output and the quality of the carbon source during vegetative growth. DLS (Figure 1**B**) and TEM (Figure 1**D**) revealed EVs in two size populations, with diverse morphologies, including vesicles bearing an outer membrane coating. The size heterogeneity strongly suggests a mixed origin for EVs.

We evaluated the purity and cargo composition of our EV preparation using LC-MS/MS (Table 1 and Supplemental Table S1). A total of 237 unique peptides corresponding to 46 unique proteins were identified. Of these, 30 proteins (65%) contain predicted signal peptides, while 16 (35%) do not.

Among the proteins containing predicted signal peptides, we identified mCherry (4% relative abundance), reflecting its expression in the XynMC11 strain used in this proteomics experiment as an EV cargo reporter. The remaining proteins included 21 enzymes (49%), 4 uncharacterized proteins (6%), and 4 proteins associated with cell wall or biofilm formation 5%).

Among the proteins lacking predicted peptides but annotated as extracellular or cell wall-associated, we detected HSP90 (3%), three cell wall remodeling enzymes (13%), four stress response proteins (9%), five uncharacterized proteins (7%), and three cytoplasmic enzymes (5%) based on Gene Ontology annotation.

Collectively, these results indicate that EV purification using a 300 kDa molecular weight cutoff filter yields highly enriched EV fractions, although trace amounts of non-EV proteins remain. This is consistent with previous LC-MS/MS analyses of EV cargo, which commonly show enrichment of cargo proteins, along with minor subsets of cytoplasmic proteins that co-purify due to cell lysis, autophagic leakage, or secretion through vacuolar or exocytic pathways 60–65.

Both A773, a wildtype reference strain, and PFI-XLN7, a strain that constitutively expresses the XlnR transcription factor and super-induces xylanase genes (*xlnA*, *xlnB*, *xlnC*, *xlnD*, and others), showed enhanced xylanase activity and EV secretion in xylose and xylan compared to glucose (Figure 2). The trend of increased EV secretion following activation suggests that post-translational mechanisms (EV biogenesis) are linked to transcriptional regulatory mechanisms. Moreover, when transcriptional activation was overdriven (PFI-XLN7), EV biogenesis was also enhanced (Figure 2). Extracellular enzymes have previously been detected in EVs from other fungi, including cellulases in *Trichoderma reesei* 61 and laccase in *C. neoformans* 66, 67.

Replacing the XlnC protein-coding region with mCherry (strain XynMC11) corroborated the observation that increased transcription enhances EV biogenesis (Figures 3 and 4). FFC data (Figure 3**E**) and fluorescence microscopy (Figure 4) confirmed that ∼30% of EVs (30.29% by FFC and 26.37% by fluorescence microscopy) carried mCherry, highlighting the selective packaging of transcriptionally induced proteins into EVs. Thus, our results strongly suggest carbon-source-dependent regulation of EV secretion and cargo loading at the post-translational level, as corroborated by others 53, 54.

Both strains XynMC11 (wildtype reference A1149 now producing mCherry) and XlnR7MC7 (XlnR overexpressing PFI-XLN7 and now overproducing mCherry) secreted mCherry-loaded EVs in media containing xylose (inducer and carbon source), but not in glucose. For example, in XynMC11, mCherry within EVs increased 34-fold, while in XlnR7MC7, mCherry within EVs increased 131-fold, suggesting that EV biogenesis demand is connected to transcriptional activation of cargo proteins (Table 2).

XynMC11 and XlnR7MC7 retain native 5’- and 3’-UTRs and the native signal peptide (Figure S2), ensuring proper targeting and trafficking of the mCherry reporter through the ER and downstream secretion pathway. The proportional increase in mCherry-containing EVs under xylose (44%) further supports the hypothesis that EV loading is dependent on the inducing carbon source and suggests that cargos are sorted into EVs via a regulated post-translational mechanism rather than passive secretion (Table 2). Others have also observed a regulatory link between transcription and post-translational processing 68–70.

The spatiotemporal analysis of mCherry localization and EV secretion (Figure 5) indicates that ER-translated protein is enhanced during early vegetative growth (Figure 5, 12 to 48 hours), accompanied by expansion of the endomembrane system that maximizes translational capacity (Figure 5, 36 to 48 hours) 71.

During this stage, ER-translated proteins are packaged into vesicles at ER exit sites via COPII coat proteins, transported through the Golgi apparatus, and directed to the hyphal tip for secretion 38, 39, 43, 72, 73.

As growth progresses, the endomembrane system undergoes pronounced expansion, with abundant mCherry-labeled cargo accumulating in intracellular compartments (Figure 5, 48–72 hours). Although the resolution of our imaging approach limits further interpretation of intracellular trafficking dynamics, it is evident that at later stages of vegetative growth (72 to 120 hours), this expanded endomembrane network generates large numbers of vesicles destined for the extracellular export.

By 72–120 hours, abundant EVs accumulate near the plasma membrane, and massive quantities of EVs are observed beyond the cell wall (Figure 5). The marked increase in secretion at this stage coincides with the stationary phase of growth (Figures 1 and 3), supporting the idea that EV release is linked to nutrient availability and contributes to continued growth under limiting conditions.

Biomass production and protein secretion involve energetic trade-offs. During exponential growth, energy primarily supports biomass production, limiting protein secretion. During the stationary phase, the energy demand for biomass decreases, allowing increased protein secretion, which peaks at approximately 72 hours. Our results show that EVs drive protein secretion, supporting this energy allocation model (see Figures 1, 2, 5, and Table 2).

The experiment shown in Figure 6 highlights the association between EVs and biofilm formation, a critical adaptive mechanism that occurs during the stationary phase and in response to nutrient scarcity. Retaining EV cargo, such as enzymes, within biofilms ensures localized nutrient acquisition and metabolic recycling. This mechanism is supported by the observed alignment between mCherry secretion and biofilm-formation kinetics, suggesting that EVs play a dual role in delivering specialized enzymes and in mediating resource conservation.

Removing the SP reduced overall EV production and mCherry incorporation into EVs, indicating that the ER, or production of ER-targeted proteins, plays a leading role in EV biogenesis (Figure 7). This finding aligns with the hypothesis that EVs originate from the ER, where newly translated cargo proteins are directly loaded into vesicles destined for extracellular secretion.

The cellular origins and functional roles of fungal EVs are intimately linked to endomembrane dynamics and are examined in this study. IVs are drivers of endomembrane metabolism and mediate the trafficking of cargo between organelles such as the ER, Golgi apparatus, and mitochondria, thereby maintaining organelle function and cellular homeostasis 46, 74–78. Many IVs originate from the ER or Golgi membranes, following the translation and modifications of mature proteins. Thus, it is plausible that fungal EVs also derive from these membranes, particularly the ER, to facilitate extracellular metabolism 45.

Our findings establish EVs as versatile vehicles for fungal adaptation, enabling them to respond to environmental signals through regulated cargo loading and secretion. This study advances our understanding of fungal EVs, providing a framework for exploring their roles in industrial applications, fungal pathogenicity, and inter-kingdom interactions. Developing a method to isolate EV-enriched fractions free of common contaminants, such as cellular debris and protein aggregates, combined with specific-fluorescence-based EV counting, offers a versatile tool with the potential to drive future discoveries. Future research should investigate the molecular mechanisms underlying EV biogenesis, cargo selection, and secretion, as well as their functional implications in diverse fungal species and ecological contexts.

## MATERIAL AND METHODS

### Chemicals and general *Aspergillus* cultivation

General chemicals, cellulosic, and hemicellulosic substrates were purchased from the best source possible: Millipore/Sigma Aldrich, MO, Megazyme, UK, and New England Biolabs (US). *A. nidulans* media and general cultivation techniques were based on 79, 80.

### Secretome measurements

Secretomes containing EVs and free-roaming proteins were evaluated using media from various culture conditions. The media were centrifuged at 12,000 ×g for 30 minutes to remove cellular debris before further analysis.

Free sugar (reducing) determinations were used in two types of experiments: (1) to determine the activity of enzymes that use a non-reducing substrate releasing reducing products (sugars), and (2) to quantify the amount of reducing sugar consumed. We used the dinitrosalicylic acid (DNS) assay developed by Sumner and Graham 81 to detect reducing sugars. The DNS-reducing sugar assay was based on the method described by Miller 82 and adapted to a microtiter dish scale. The DNS reagent we used contained 0.75% dinitrosalicylic acid, 0.5% phenol, 0.5% sodium metabisulfite, 1.4% sodium hydroxide, 21% sodium, and potassium tartrate.

Xylanase activity was determined using beechwood hemicellulose as the substrate, and activity was measured by the release of reducing sugars that react with DNS 82. Briefly, 10–50 μ L of extracellular protein extract was added to a mixture of 300 μ L of 1% beechwood xylan and 50 mM ammonium acetate buffer, and reactions were incubated for 10, 20, or 30 min at 45 ${}^{\circ }$ C before the addition of 300 μ L of DNS. Control reactions, blanks that determine the presence of reducing sugars in the starting mixture, contained all the same reagents except that DNS was added before the enzyme sample. The amount of reducing sugar produced during the enzyme-catalyzed reaction was calculated by subtracting the control (blanks) ABS 540 nm from the enzyme reaction ABS 540 nm and the net gain in ABS 540 nm converted into enzyme units ( μ mol/min/ μ g protein).

mCherry accumulation in secretomes was measured by mCherry fluorescence emission using a BioTek Synergy MX Multi-Mode microplate reader (monochromator-based system) with fluorescent capabilities. Fluorescence intensities were captured using a Xenon flash lamp and a red-shifted photomultiplier tube (PMT) detector with the plate reader. mCherry amounts are reported as relative fluorescence units (RFUs), dimensionless values, set up for excitation at 587 nanometers and emission at 610 nanometers. Appropriate blanks, minimal media, or 1X PBS were used in all RFU measurements and subtracted from the RFU readings taken from the samples.

Total protein concentration in the secretome was quantified using a microtiter plate-based assay following the Bradford method 83, 84. A Bio-Rad protein assay reagent (Bio-Rad Laboratories, USA) was employed, with bovine serum albumin as the standard. Absorbance was measured at 595 nm using a Tecan Infinite M200 UV-Vis 96-well plate reader (Tecan, Männedorf, Switzerland).

To follow our vegetative growth dynamics in liquid-cultured media, we measured the dry weight of harvested vegetative mycelial mats from liquid cultures, dried at 68 ${}^{\circ }$ C, and reported as milligrams per mycelium.

### Biofilm production

The formation of a biofilm was visualized by inoculating *A. nidulans* conidia onto coverslips at the bottom of a 60 × 15 mm Petri plate containing liquid minimal media, as described by Shay *et al.* 57, with some slight modifications. Following incubation at 30 ${}^{\circ }$ C on coverslips, the coverslips were washed with 1X PBS (pH 7.4) and stained with Calcofluor White to detect cells and the biofilm. The coverslips were then inverted onto microscope slides and viewed under a Nikon Eclipse Ti2 fluorescence microscope (described in “Microscopy” methodology) using filter cubes for Texas Red (to detect mCherry) and DAPI (to detect the Calcofluor).

In addition, biofilm production was quantified using a Crystal Violet (CV) assay, following the protocol described by Lin *et al.* 58, with slight modifications. Conidia were inoculated into a 6-well microtiter plate containing 3 mL of liquid media with a 1% carbon source in each well and incubated at 37 ${}^{\circ }$ C for 72 hours. Mycelia and media were removed from the wells and washed with 1X PBS (pH 7.4). Wells were stained with 0.2% (w/v) CV for 10 minutes and then washed with distilled H ${}_{2}$ O. 30% (v/v) acetic acid was used to dissolve the remaining CV, and absorbance (OD at 560 nm) was measured using a Tecan Infinite M200 UV-Vis 96-well plate reader 58.

### Liquid growth conditions optimized for EV production

*A. nidulans* strain A773 (*pyrG89, wA3, pyroA4*) and A1149 (*pyrG89, pyroA4,* Δ *nkuA::argB*) were from the Fungal Genetics Stock Center (FGSC, Kansas State University, Manhattan, Kansas) and characterized the wildtype reference state in physiological and biochemical experiments. Both strains also served as the original hosts for generating all genetically modified constructs reported in this study.

For liquid medium culturing on Petri dishes with slippable lids (60 mm × 15 mm), a uniform inoculum of 10–50 μ L of a fungal spore stock suspension (1 × 10 ${}^{8}$ /mL) was directly pipetted onto plates containing 10 mL minimal medium (MM) 85 supplemented with the required auxotrophic requirements for each strain and 1% of a suitable carbon source, glucose (Fisher Scientific), xylose (Sigma Aldrich), glycerol (Fisher Scientific) and xylan (from birchwood; Sigma Aldrich). Inoculated Petri dishes were incubated at 37 ${}^{\circ }$ C in a stationary position for two or more days, as indicated.

### Isolation, purification, detection, and EV quantification

EVs were purified from 3 mL samples (or as otherwise stated), carefully collected from stationary liquid growth plates with minimal disruption of the mycelial mat, and then centrifuged at 12,000 × g for 10 minutes to obtain a cell-free supernatant (CFS). The CFS was further subjected to 10,000 × g ultrafiltration (300 kDa cutoff, Vivaspin 500, Sartorius). The retentate CFS was washed once with 500 μ L of 1X PBS (phosphate-buffered saline containing 100 mM NaCl) and stored at 4 ${}^{\circ }C$ until further analysis.

For EV detection, we used Dynamic Light Scattering (Zetasizer ZS, Malvern) and Transmission Electron Microscopy (JEM-2100, JEOL) on purified EV samples.

To quantify EVs, we used an Acea NovoCyte Flow Cytometer (12 fluorescence channels, 3 laser system) and a fluorescent membrane stain, CellBrite 488 (Biotium, San Francisco, CA), to count purified EVs and EVs containing mCherry. CellBrite 488 is a membrane stain that binds to lipid bilayers of plasma membranes and vesicles, making it useful for detecting (microscopy) and tracking (FFC) EVs based on their membrane structure. 100 μ L of purified EVs were combined with 10 μ L of CellBrite488, and the mixture was counted with a threshold of 1,000 events and gating optimized to match the predicted size constraints. Fluorescein isothiocyanate (FITC-ex. 498 nm, em. 517 nm) was used for CellBrite488’s positive control, and Texas Red (ex. 596 nm, em. 615 nm) was used as mCherry’s positive control. A negative control (unstained sample) was also used, and all measurements were taken in triplicate 86.

### Mass spectrometry (LC-MS/MS)

#### Protein sample preparation

To analyze the potential cargoes of EVs, we grew strain XynMC11 in xylose for 72 hours and collected 250 mL of medium for EV purification. Purified EV-derived proteins were reduced with 10 mM TCEP (RT, 1 h) and alkylated with 10 mM iodoacetamide (dark, RT, 1 h). Samples were digested with trypsin (Promega V5072) at an enzyme-to-substrate ratio of ∼1:25 (w/v) overnight at 37 ${}^{\circ }$ C in 25 mM ammonium bicarbonate (pH 8.0).

#### Sample cleanup and nanoLC

Digested peptides were acidified with 0.5% trifluoroacetic acid (TFA), desalted using C18 solid-phase extraction (SPE) pipette tips according to Agilent instructions, eluted in 0.1% formic acid/60% acetonitrile, dried, and reconstituted in 0.1% formic acid.

#### LC-MS/MS

Samples were injected onto a 75 μ m × 50 cm PepMan C18 column (2 μ m, Thermo Fisher); separated with a 60 min linear gradient at 250 nL/min; ionized *via* Nanospray Flex (1.9 kV, 300 ${}^{\circ }$ C); analyzed on Orbitrap Fusion with MS1 at 120 k resolution (375–1575 m/z, AGC 4e5, 50 ms); high speed DDA top-speed 5 s; MS/MS in ion trap (rapid scan, AGC 5e4, dynamic injection times, HCD 32%, dynamic exclusion 45 s, charge ＋2 to ＋6).

#### Data Analysis

Each sample was analyzed twice by LC-MS/MS and processed as a single sample. Spectra were processed with MaxQuant v2.0.1.0 87, searched against a complete genome *A. nidulans* protein database from NCBI; carbamidomethyl as fixed, oxidation (M) variable; 4.5ppm precursor and 20ppm fragment tolerances; 2 missed cleavages; FDR<1%; annotate IDs with Python and UniProt mapping.

### Construction of the EV-cargo reporter strain XynMC11 and derivatives

Figure S2 illustrates the AN1818 chromosomal region, showing the coordinates and the genetic elements that are subject to molecular modifications. Figure S2B depicts the sizes (not to scale) of PCR-amplified DNA fragments (F1-F6) and the corresponding template DNA sources: FGSC *A. nidulans* strains A1149 and A234, the pFTK049 plasmid containing mCherry (ADDGENE), and the pUC19 vector. Fragments F1-F6 were assembled using HiFi DNA Assembly Master Mix (NebBuilder). A single DNA fragment, consisting of the assembled F1-F5 fragments, was PCR-amplified using *Escherichia coli* purified plasmids as a template and transformed into *A. nidulans* A1149 (*pyrG89, pyroA4,* Δ *kuA::argB*). Figure S2C shows the expected genotype of recombinants arising from a double crossover integration at the AN1818 locus. To generate a strain lacking the signal peptide (SP), PCR fusion primers were used to amplify the entire pBP6 plasmid (Figure S2C), excluding the SP region. Ligation of the PCR product resulted in plasmid pBP2, which was transformed into A1149 to produce the strain Δ XynSP (Figure S2D).

Recovered transformants were selected and plated on master plates. Validation was performed by PCR amplification of *A. nidulans* genomic DNA (see miniprep protocol below) using multiple primers designed to amplify at least two fused fragments internal to the AN1818 locus, confirming the replacement of *xlnC* with *mCherry*. For selected recombinants XynCMC11 and Δ XynSP, the chromosomal region was further PCR amplified using primers VFxynUP and VFxynDWr, which anneal ∼900 bp upstream and downstream of the AN1818 locus, respectively. The resulting ∼7,000 bp PCR products were fully sequenced (Plasmidsaurus Eugene, OR) and confirmed the reliability of our construct with no obvious mutations.

To construct strain XlnR7MC7, which constitutively expresses XlnR 51, strain XynCMC11 (*pyrG89*, *pyroA4*, Δ *kuA::argB*, Δ *xlnC::mCherry*) was crossed with strain PFI-XLN7 (*paba::gpdA::XlnR::paba*, *pyroA4*) using standard sexual crossing techniques 85. Offspring were screened, and strain XlnR7MC7 (*paba::gpdA::XlnR::paba*, *pyroA4*, Δ *xlnC::mCherry*) was selected. This strain overexpresses mCherry under the control of XlnR.

### *A. nidulans* transformation

followed the reference protocol by Szewczyk *et al.* 88, with a few modifications: a) 10 mL of 2X protoplasting solution contained 100 mg of VinoTaste Pro (Novozymes Bagsvaerd, Denmark), 50 mg of lysozyme (from chicken egg, Sigma Aldrich), 820 mg KCl, 190 mg citric acid, pH 5.8, and b) after protoplasting the mixture was centrifuged at 500 × g for 15 minutes and the supernatant containing the protoplasts transferred to a new 50 mL conical tube followed by centrifugation at 2,500 × g for 10 minutes. The pelleted protoplasts were resuspended with a 1 mL pipette, topped with 40 mL of STC50, and centrifuged at 2,500 × g for 10 minutes. Protoplasts were resuspended in a final volume of 1 mL with STC50 and used for transformations 88.

### Miniprep DNA extraction

To extract genomic DNA from transformants, we used a modified miniprep method adapted from several published protocols 89–91. The process utilizes an “all-in-one tube concept”—growing a small vegetative mycelium in 250 μ L of 1X growth medium with an overnight incubation at 250 rpm at 37 ${}^{\circ }$ C. Next day, add 250 μ L of 2X Lysis buffer 1 (100 mM Tris/HCl pH 7.5, 200 mM NaCl, and 5.0 mg/mL of lysozyme (from chicken egg Millipore/Sigma Aldrich, MO), incubate stationary at 50°C for 15 minutes, then add 250 μ L of 3X Lysis buffer 2 (150 mM Tris/HCl, pH 7.5, 150 mM EDTA, 3% SDS) and incubate stationary for another 15 minutes at 70°C. Centrifuge for 5 minutes at 10,000 × g and transfer 450 μ L of SN to a new tube with 900 μ L of 95% EtOH. Centrifuge for 5 minutes at 10,000 × g and wash the pellet with 70% EtOH once before drying at 68 ${}^{\circ }$ C for 5 minutes. Resuspend the pellet in 20 μ L of TE (10 mM Tris/HCl, pH 7.5, 1 mM EDTA) and use 1 μ L as a template for regular PCR reactions and 5 μ L for PCR products longer than 6 kb.

### Microscopy

#### Fluorescence microscopy

Isolated EVs stored in 1X PBS (pH 7.4) were re-concentrated using a 300 kDa filter and stained with CellBrite 488 membrane stain. Ten microliters of the concentrated and stained EVs were placed on a clean microscope slide and visualized using the fluorescence microscope described below. *A. nidulans* cells were prepared for microscopy by forming a small, rectangular agar pad containing a 1% carbon source and 0.1% (w/v) Calcofluor White on a microscope slide. Conidia were diluted 1:1000 in sterile water, inoculated onto the agar pad, and incubated until the desired growth time point. For incubation, slides were placed on a bent glass rod at the bottom of a Petri dish, and 3 mL of distilled H ${}_{2}$ O was added to maintain humidity. After incubation, an adhesive frame (Gene Frame, 1.5 × 1.6 cm, Thermo Fisher Scientific) was placed around the agar pad to prevent desiccation, and a coverslip was placed over the sample for imaging.

Fluorescence microscopy imaging was performed using Nikon Eclipse Ti or Ti2 inverted microscopes with Photometrics Prime 95B sCMOS camera, 100x Plan Apo oil objectives (NA 1.45, Nikon), and LED-based (Xylis X-Cite or Lumencor SOLA 365) fluorescent illumination systems. Microscopy images were captured using the NIS-Elements AR 5.11.03 64-bit software. Filter cubes for Texas Red (to detect mCherry) and GFP (to detect CellBrite 488) were used for isolated EV images, and filter cubes for Texas Red and DAPI (to detect Calcofluor White) were used for live-cell imaging. All filter cubes captured images with 33% power and exposures of 200–400 milliseconds.

#### Confocal microscopy

Confocal microscopy with live-cell imaging was performed using a Zeiss LSM980 with 36 simultaneous channels, equipped with lasers specifically designed for Calcofluor White and mCherry. Images were captured and processed using Zen Blue (3.3). Overlays of all micrographs were created using ImageJ2 (v. 2.14.0/1.54f).

#### Transmission Electron Microscopy

Isolated EVs stored in 1X PBS were subjected to TEM imaging using a JEM-2100 TEM (JEOL). For TEM analysis of hyphal cross-sections, *A. nidulans* was grown in a 60 × 15 mm Petri dish with a liquid minimal medium for 48 hours. The resulting mycelial mat was cut into several pieces, approximately 1 cm ${}^{3}$ , and placed into 2% buffered glutaraldehyde (pH 7.0) for tissue fixation. The tissue samples were washed in buffer and then fixed in 1% aqueous osmium tetroxide (OsO ${}_{4}$ ). The mycelia were rewashed in buffer and dehydrated by increasing ethanol concentrations before being soaked in propylene oxide and placed in a poly/bed 812 epoxy resin overnight. The tissues were then embedded in an embedding medium and dried at 60 ${}^{\circ }$ C before being sliced into thin sections with an ultramicrotome.

## AUTHOR CONTRIBUTIONS

REP planned and implemented vesicle extraction protocols. Developed dynamic light scattering (DSL) and flow cytometry (FFC) detection protocols, performed most experiments with microscopy, and analyzed the data. LW was instrumental in most microscopic analyses, performed TEMs, and assisted with confocal microscopy. PB suggested the XLN7 overexpression strain, critically read the draft manuscript, and provided funding for consumables. RAP supervised the experiments, data analysis, and reviewed/edited the last version of the manuscript.

## SUPPLEMENTAL MATERIAL

All supplemental data for this article are available online at http://microbialcell.com/researcharticles/2026a-pope-microbial-cell/. 

## CONFLICT OF INTERESTS STATEMENT

The authors declare that they have no known competing commercial interests or personal relationships that could have influenced the work reported in this paper.

## ABBREVIATIONS

CLS – cell-free supernatant

DLS – dynamic light scattering

ER – endoplasmic reticulum

EVs – extracellular vesicles

IVs – intracelluar vesicles

MVBs – multivesicular bodies

SP – signal peptide

SV – secretory vesicle

TEM – transmission electron microscopy

FFC – fluorescence flow cytometry

## References

[b00da2] Naranjo-Ortiz M.A., Gabaldon T. (2019). Fungal evolution: major ecological adaptations and evolutionary transitions. Biol Rev Camb Philos Soc.

[b00e0b] Chang Y., Wang Y., Mondo S., Ahrendt S., Andreopoulos W., Barry K., Beard J., Benny G.L., Blankenship S., Bonito G., Cuomo C., Desiro A., Gervers K.A., Hundley H., Kuo A., LaButti K., Lang B.F., Lipzen A., O’Donnell K., Pangilinan J., Reynolds N., Sandor L., Smith M.E., Tsang A., Grigoriev I.V., Stajich J.E., Spatafora J.W. (2022). Evolution of zygomycete secretomes and the origins of terrestrial fungal ecologies. iScience.

[b00fb4] Heaton L.L.M., Jones N.S., Fricker M.D. (2020). A mechanistic explanation of the transition to simple multicellularity in fungi. Nat Commun.

[b01027] Pellegrin C., Morin E., Martin F.M., Veneault-Fourrey C. (2015). Comparative analysis of secretomes from ectomycorrhizal fungi with an emphasis on small-secreted proteins. Front Microbiol.

[b010a4] Keller N.P. (2019). Fungal secondary metabolism: regulation, function and drug discovery. Nat Rev Microbiol.

[b01100] Kunzler M. (2018). How fungi defend themselves against microbial competitors and animal predators. PLoS Pathog.

[b01157] Bahram M., Netherway T. (2022). Fungi as mediators linking organisms and ecosystems. FEMS Microbiol Rev.

[b011bd] Girard V., Dieryckx C., Job C., Job D. (2013). Secretomes: the fungal strike force. Proteomics.

[b01259] Liu D., Li J., Zhao S., Zhang R., Wang M., Miao Y., Shen Y., Shen Q. (2013). Secretome diversity and quantitative analysis of cellulolytic Aspergillus fumigatus Z5 in the presence of different carbon sources. Biotechnol Biofuels.

[b0130d] Saykhedkar S., Ray A., Ayoubi-Canaan P., Hartson S.D., Prade R., Mort A.J. (2012). A time course analysis of the extracellular proteome of Aspergillus nidulans growing on sorghum stover. Biotechnol Biofuels.

[b013a7] Tsang A., Butler G., Powlowski J., Panisko E.A., Baker S.E. (2009). Analytical and computational approaches to define the Aspergillus niger secretome. Fungal Genet Biol.

[b01437] Eichelbaum K., Winter M., Berriel Diaz M., Herzig S., Krijgsveld J. (2012). Selective enrichment of newly synthesized proteins for quantitative secretome analysis. Nat Biotechnol.

[b014c7] McCotter S.W., Horianopoulos L.C., Kronstad J.W. (2016). Regulation of the fungal secretome. Curr Genet.

[b0153d] Schultzhaus Z.S., Shaw B.D. (2015). Endocytosis and exocytosis in hyphal growth. Fungal Biol Rev.

[b015a6] Albuquerque P.C., Nakayasu E.S., Rodrigues M.L., Frases S., Casadevall A., Zancope-Oliveira R.M., Almeida I.C., Nosanchuk J.D. (2008). Vesicular transport in Histoplasma capsulatum: an effective mechanism for trans-cell wall transfer of proteins and lipids in ascomycetes. Cell Microbiol.

[b0165d] Casadevall A., Nosanchuk J.D., Williamson P., Rodrigues M.L. (2009). Vesicular transport across the fungal cell wall. Trends Microbiol.

[b016e0] Rodrigues M.L., Nimrichter L., Oliveira D.L., Nosanchuk J.D., Casadevall A. (2008). Vesicular trans-cell wall transport in fungi: A mechanism for the delivery of virulence-associated macromolecules?. Lipid Insights.

[b0176d] Nimrichter L., de Souza MM., Del Poeta M., Nosanchuk J.D., Joffe L., Tavares Pde M., Rodrigues M.L. (2016). Extracellular vesicle-associated transitory cell wall components and their impact on the interaction of fungi with host cells. Front Microbiol.

[b01811] Rizzo J., Chaze T., Miranda K., Roberson R.W., Gorgette O., Nimrichter L., Matondo M., Latge J.P., Beauvais A., Rodrigues M.L. (2020). Characterization of extracellular vesicles produced by Aspergillus fumigatus protoplasts. mSphere.

[b018df] Rodrigues M.L., Janbon G., O’Connell R.J., Chu T.T., May R.C., Jin H., Reis F.C.G., Alves L.R., Puccia R., Fill T.P., Rizzo J., Zamith-Miranda D., Miranda K., Goncalves T., Ene I.V., Kabani M., Anderson M., Gow N.A.R., Andes D.R., Casadevall A., Nosanchuk J.D., Nimrichter L. (2025). Characterizing extracellular vesicles of human fungal pathogens. Nat Microbiol.

[b01a4c] Gibson R.K., Peberdy J.F. (1972). Fine structure of protoplasts of Aspergillus nidulans. J Gen Microbiol.

[b01ab5] Rizzo J., Rodrigues M.L., Janbon G. (2020). Extracellular vesicles in fungi: Past, present, and future perspectives. Front Cell Infect Microbiol.

[b01b23] Takeo K., Uesaka I., Uehira K., Nishiura M. (1973). Fine structure of Cryptococcus neoformans grown in vitro as observed by freeze-etching. J Bacteriol.

[b01ba6] Pathan M., Fonseka P., Chitti S.V., Kang T., Sanwlani R., Van Deun J., Hendrix A., Mathivanan S. (2019). Vesiclepedia 2019: a compendium of RNA, proteins, lipids and metabolites in extracellular vesicles. Nucleic Acids Res.

[b01c5d] da Peres Silva R., Puccia R., Rodrigues M.L., Oliveira D.L., Joffe L.S., Cesar G.V., Nimrichter L., Goldenberg S., Alves L.R. (2015). Extracellular vesicle-mediated export of fungal RNA. Sci Rep.

[b01d1b] Rodrigues M.L., Casadevall A. (2018). A two-way road: novel roles for fungal extracellular vesicles. Mol Microbiol.

[b01d84] Palade G. (1975). Intracellular aspects of the process of protein synthesis. Science.

[b01de0] Vitale A., Denecke J. (1999). The endoplasmic reticulum-gateway of the secretory pathway. Plant Cell.

[b01e49] Bednarek S.Y., Ravazzola M., Hosobuchi M., Amherdt M., Perrelet A., Schekman R., Orci L. (1995). COPI- and COPII-coated vesicles bud directly from the endoplasmic reticulum in yeast. Cell.

[b01ef3] Brandizzi F., Barlowe C. (2013). Organization of the ER-Golgi interface for membrane traffic control. Nat Rev Mol Cell Biol.

[b01f5c] Cai H., Reinisch K., Ferro-Novick S. (2007). Coats, tethers, Rabs, and SNAREs work together to mediate the intracellular destination of a transport vesicle. Dev Cell.

[b01fd2] Pantazopoulou A. (2016). The Golgi apparatus: insights from filamentous fungi. Mycologia.

[b0202e] Pinar M., Pantazopoulou A., Penalva M.A. (2013). Live-cell imaging of Aspergillus nidulans autophagy: RAB1 dependence, Golgi independence and ER involvement. Autophagy.

[b020a4] Bartnicki-Garcia S., Heath I.B. (1990). Role of vesicles in apical growth and a new mathematical model of hyphal morphogenesis. Tip Growth in Plant and Fungal Cells.

[b0210c] Harris S.D., Read N.D., Roberson R.W., Shaw B., Seiler S., Plamann M., Momany M. (2005). Polarisome meets spitzenkorper: microscopy, genetics, and genomics converge. Eukaryot Cell.

[b021b6] Martzoukou O., Diallinas G., Amillis S. (2018). Secretory vesicle polar sorting, endosome recycling and cytoskeleton organization require the AP-1 complex in aspergillus nidulans. Genetics.

[b0222c] Riquelme M., Aguirre J., Bartnicki-Garcia S., Braus G.H., Feldbrugge M., Fleig U., Hansberg W., Herrera-Estrella A., Kamper J., Kuck U., Mourino-Perez R.R., Takeshita N., Fischer R. (2018). Fungal morphogenesis, from the polarized growth of hyphae to complex reproduction and infection structures. Microbiol Mol Biol Rev.

[b02321] Riquelme M., Bartnicki-Garcia S., Gonzalez-Prieto J.M., Sanchez-Leon E., Verdin-Ramos J.A., Beltran-Aguilar A., Freitag M. (2007). Spitzenkorper localization and intracellular traffic of green fluorescent protein-labeled CHS-3 and CHS-6 chitin synthases in living hyphae of Neurospora crassa. Eukaryot Cell.

[b023cb] Steinberg G. (2007). Hyphal growth: a tale of motors, lipids, and the Spitzenkorper. Eukaryot Cell.

[b02427] Riquelme M., Sanchez-Leon E. (2014). The Spitzenkorper: a choreographer of fungal growth and morphogenesis. Curr Opin Microbiol.

[b0248d] Chevalier L., Pinar M., Le Borgne R., Durieu C., Penalva M.A., Boudaoud A., Minc N. (2023). Cell wall dynamics stabilize tip growth in a filamentous fungus. PLoS Biol.

[b02532] Schuster M., Martin-Urdiroz M., Higuchi Y., Hacker C., Kilaru S., Gurr S.J., Steinberg G. (2016). Co-delivery of cell-wall-forming enzymes in the same vesicle for coordinated fungal cell wall formation. Nat Microbiol.

[b025d9] Hayakawa Y., Ishikawa E., Shoji J.Y., Nakano H., Kitamoto K. (2011). Septum-directed secretion in the filamentous fungus Aspergillus oryzae. Mol Microbiol.

[b02669] Demos E., Dimou S., Scazzocchio C., Diallinas G. (2024). Screens for mutants defective in UapA trafficking highlight the importance of ER-exit as a primary control point in transporter biogenesis. Fungal Genet Biol.

[b026e4] Dimou S., Dionysopoulou M., Sagia G.M., Diallinas G. (2022). Golgi-bypass is a major unconventional route for translocation to the plasma membrane of non-apical membrane cargoes in aspergillus nidulans. Front Cell Dev Biol.

[b0275f] Dimou S., Martzoukou O., Dionysopoulou M., Bouris V., Amillis S., Diallinas G. (2020). Translocation of nutrient transporters to cell membrane via Golgi bypass in Aspergillus nidulans. EMBO Rep.

[b027f9] Read N.D. (2011). Exocytosis and growth do not occur only at hyphal tips. Mol Microbiol.

[b02855] Stricker A.R., Mach R.L., de Graaff LH. (2008). Regulation of transcription of cellulases- and hemicellulases-encoding genes in Aspergillus niger and Hypocrea jecorina (Trichoderma reesei). Appl Microbiol Biotechnol.

[b028cb] Wu V.W., Thieme N., Huberman L.B., Dietschmann A., Kowbel D.J., Lee J., Calhoun S., Singan V.R., Lipzen A., Xiong Y., Monti R., Blow M.J., O’Malley R.C., Grigoriev I.V., Benz J.P., Glass N.L. (2020). The regulatory and transcriptional landscape associated with carbon utilization in a filamentous fungus. Proc Natl Acad Sci U S A.

[b029ea] Ballmann P., Lightfoot J., Muller M., Droge S., Prade R. (2019). Redesigning the Aspergillus nidulans xylanase regulatory pathway to enhance cellulase production with xylose as the carbon and inducer source. Microb Cell Fact.

[b02a77] M. Flipphi, van de Vondervoort PJ, Ruijter GJ, Visser J., Arst H.N. Jr., Felenbok B. (2003). Onset of carbon catabolite repression in Aspergillus nidulans. Parallel involvement of hexokinase and glucokinase in sugar signaling. J Biol Chem.

[b02b14] Oliveira D.L., Nakayasu E.S., Joffe L.S., Guimaraes A.J., Sobreira T.J., Nosanchuk J.D., Cordero R.J., Frases S., Casadevall A., Almeida I.C., Nimrichter L., Rodrigues M.L. (2010). Characterization of yeast extracellular vesicles: evidence for the participation of different pathways of cellular traffic in vesicle biogenesis. PLoS One.

[b02bfc] Zarnowski R., Sanchez H., Covelli A.S., Dominguez E., Jaromin A., Bernhardt J., Mitchell K.F., Heiss C., Azadi P., Mitchell A., Andes D.R. (2018). Candida albicans biofilm-induced vesicles confer drug resistance through matrix biogenesis. PLoS Biol.

[b02cd5] Morelli K.A., Kerkaert J.D., Cramer R.A. (2021). Aspergillus fumigatus biofilms: Toward understanding how growth as a multicellular network increases antifungal resistance and disease progression. PLoS Pathog.

[b02d46] Peiqian L., Xiaoming P., Huifang S., Jingxin Z., Ning H., Birun L. (2014). Biofilm formation by Fusarium oxysporum f. sp. cucumerinum and susceptibility to environmental stress. FEMS Microbiol Lett.

[b02de3] Shay R., Wiegand A.A., Trail F. (2022). Biofilm formation and structure in the filamentous fungus fusarium graminearum, a plant pathogen. Microbiol Spectr.

[b02e54] Lin C.J., Hou Y.H., Chen Y.L. (2020). The histone acetyltransferase GcnE regulates conidiation and biofilm formation in Aspergillus fumigatus. Med Mycol.

[b02eca] Owji H., Nezafat N., Negahdaripour M., Hajiebrahimi A., Ghasemi Y. (2018). A comprehensive review of signal peptides: Structure, roles, and applications. Eur J Cell Biol.

[b02f5a] Bleackley M.R., Dawson C.S., Anderson M.A. (2019). Fungal extracellular vesicles with a focus on proteomic analysis. Proteomics.

[b02fcb] de Paula RG., Antonieto A.C.C., Nogueira K.M.V., Ribeiro L.F.C., Rocha M.C., Malavazi I., Almeida F., Silva R.N. (2019). Extracellular vesicles carry cellulases in the industrial fungus Trichoderma reesei. Biotechnol Biofuels.

[b0307f] Gil-Bona A., Llama-Palacios A., Parra C.M., Vivanco F., Nombela C., Monteoliva L., Gil C. (2015). Proteomics unravels extracellular vesicles as carriers of classical cytoplasmic proteins in Candida albicans. J Proteome Res.

[b03129] Rodrigues M.L., Nakayasu E.S., Almeida I.C., Nimrichter L. (2014). The impact of proteomics on the understanding of functions and biogenesis of fungal extracellular vesicles. J Proteomics.

[b031a9] Silva G.R., de Pina Cavalcanti F., Melo R.M., Cintra E., Lima E.M., Hamann P.R.V., do Vale LHF., Ulhoa C.J., Almeida F., Noronha E.F. (2024). Extracellular vesicles from the mycoparasitic fungus Trichoderma harzianum. Antonie Van Leeuwenhoek.

[b03277] Vallejo M.C., Nakayasu E.S., Matsuo A.L., Sobreira T.J., Longo L.V., Ganiko L., Almeida I.C., Puccia R. (2012). Vesicle and vesicle-free extracellular proteome of Paracoccidioides brasiliensis: comparative analysis with other pathogenic fungi. J Proteome Res.

[b0332e] Panepinto J., Komperda K., Frases S., Park Y.D., Djordjevic J.T., Casadevall A., Williamson P.R. (2009). Sec6-dependent sorting of fungal extracellular exosomes and laccase of Cryptococcus neoformans. Mol Microbiol.

[b033d8] Park Y.D., Chen S.H., Camacho E., Casadevall A., Williamson P.R. (2020). Role of the ESCRT pathway in laccase trafficking and virulence of Cryptococcus neoformans. Infect Immun.

[b03468] Kwon M.J., Arentshorst M., Fiedler M., de Groen FLM., Punt P.J., Meyer V., Ram AFJ. (2014). Molecular genetic analysis of vesicular transport in Aspergillus niger reveals partial conservation of the molecular mechanism of exocytosis in fungi. Microbiology.

[b03512] Kwon S., Tisserant C., Tulinski M., Weiberg A., Feldbrügge M. (2020). Inside-out: from endosomes to extracellular vesicles in fungal RNA transport. Fungal Biol Rev.

[b035a2] Penalva M.A., Moscoso-Romero E., Hernandez-Gonzalez M. (2020). Tracking exocytosis of a GPI-anchored protein in Aspergillus nidulans. Traffic.

[b03618] Higuchi Y. (2021). Membrane traffic related to endosome dynamics and protein secretion in filamentous fungi. Biosci Biotechnol Biochem.

[b03674] Pinar M., Penalva M.A. (2021). The fungal RABOME: RAB GTPases acting in the endocytic and exocytic pathways of Aspergillus nidulans (with excursions to other filamentous fungi). Mol Microbiol.

[b036dd] Yang S., Zhou X., Guo P., Lin Y., Fan Q., Zuriegat Q., Lu S., Yang J., Yu W., Liu H., Lu G., Shim W.B., Wang Z., Yun Y. (2021). The exocyst regulates hydrolytic enzyme secretion at hyphal tips and septa in the banana fusarium wilt fungus Fusarium odoratissimum. Appl Environ Microbiol.

[b037dd] Bielska E., May R.C. (2019). Extracellular vesicles of human pathogenic fungi. Curr Opin Microbiol.

[b03843] Dimou S., Georgiou X., Sarantidi E., Diallinas G., Anagnostopoulos A.K. (2021). Profile of membrane cargo trafficking proteins and transporters expressed under N source derepressing conditions in Aspergillus nidulans. J Fungi.

[b038d0] Menke J., Weber J., Broz K., Kistler H.C. (2013). Cellular development associated with induced mycotoxin synthesis in the filamentous fungus Fusarium graminearum. PLoS One.

[b03950] Tang B.L., Wang Y., Ong Y.S., Hong W. (2005). COPII and exit from the endoplasmic reticulum. Biochim Biophys Acta.

[b039d3] Tu Y., Zhao L., Billadeau D.D., Jia D. (2020). Endosome-to-TGN trafficking: organelle-vesicle and organelle-organelle interactions. Front Cell Dev Biol.

[b03a50] Pontecorvo G. (1969). Genetic analysis of “somatic” cells in filamentous fungi. Wistar Inst Symp Monogr.

[b03aa6] Pontecorvo G., Roper J.A., Hemmons L.M., Macdonald K.D., Bufton A.W. (1953). The genetics of Aspergillus nidulans. Adv Genet.

[b03b33] Sumner J.B., Graham V.A. (1921). Dinitrosalicylic acid: A reagent for the estimation of sugar in normal and diabetic urine. J Biol Chem.

[b03b9c] Miller G.L. (1959). Use of dinitrosalicylic acid reagent for determination of reducing sugar. Anal Chem.

[b03bf8] Bradford M.M. (1976). A rapid and sensitive method for the quantitation of microgram quantities of protein utilizing the principle of protein-dye binding. Anal Biochem.

[b03c51] Marshall T., Williams K.M. (1992). Coomassie blue protein dye-binding assays measure formation of an insoluble protein-dye complex. Anal Biochem.

[b03cba] Clutterbuck A.J. (1992). Sexual and parasexual genetics of Aspergillus species. Biotechnology.

[b03d13] Gorgens A., Bremer M., Ferrer-Tur R., Murke F., Tertel T., Horn P.A., Thalmann S., Welsh J.A., Probst C., Guerin C., Boulanger C.M., Jones J.C., Hanenberg H., Erdbrugger U., Lannigan J., Ricklefs F.L., El-Andaloussi S., Giebel B. (2019). Optimisation of imaging flow cytometry for the analysis of single extracellular vesicles by using fluorescence-tagged vesicles as biological reference material. J Extracell Vesicles.

[b03e48] Tyanova S., Temu T., Carlson A., Sinitcyn P., Mann M., Cox J. (2015). Visualization of LC-MS/MS proteomics data in MaxQuant. Proteomics.

[b03ee5] Szewczyk E., Nayak T., Oakley C.E., Edgerton H., Xiong Y., Taheri-Talesh N., Osmani S.A., Oakley B.R. (2006). Fusion PCR and gene targeting in Aspergillus nidulans. Nat Protoc.

[b03f9c] Chomczynski P., Rymaszewski M. (2006). Alkaline polyethylene glycol-based method for direct PCR from bacteria, eukaryotic tissue samples, and whole blood. Biotechniques.

[b04002] Fraczek M.G., Zhao C., Dineen L., Lebedinec R., Bowyer P., Bromley M., Delneri D. (2019). Fast and reliable PCR amplification from Aspergillus fumigatus spore suspension without traditional DNA extraction. Curr Protoc Microbiol.

[b040aa] Walch G., Knapp M., Rainer G., Peintner U. (2016). Colony-PCR is a rapid method for DNA amplification of hyphomycetes. J Fungi.

