# Supplementary material for: Regulation of extracellular vesicles for protein secretion in Aspergillus nidulans
Source: Microb Cell. 2026 Jan 28;13:28–43. doi: 10.15698/mic2026.01.866 (PMC12867487; doi:10.15698/mic2026.01.866)
Supplement: Supplementary file 1 [file mic-13-028-s01.pdf]

Supplemental

Regulation of Extracellular Vesicles for Protein Secretion in *Aspergillus nidulans*

Rebekkah E. Pope, Patrick Ballmann, Lisa Whitworth and Rolf A. Prade
Department of Microbiology & Molecular Genetics & OSU Microscopy Laboratory, Oklahoma State University, Stillwater OK, and Prüf- und Forschungsinstitut Pirmasens e.V., Pirmasens, Germany

Extended Data

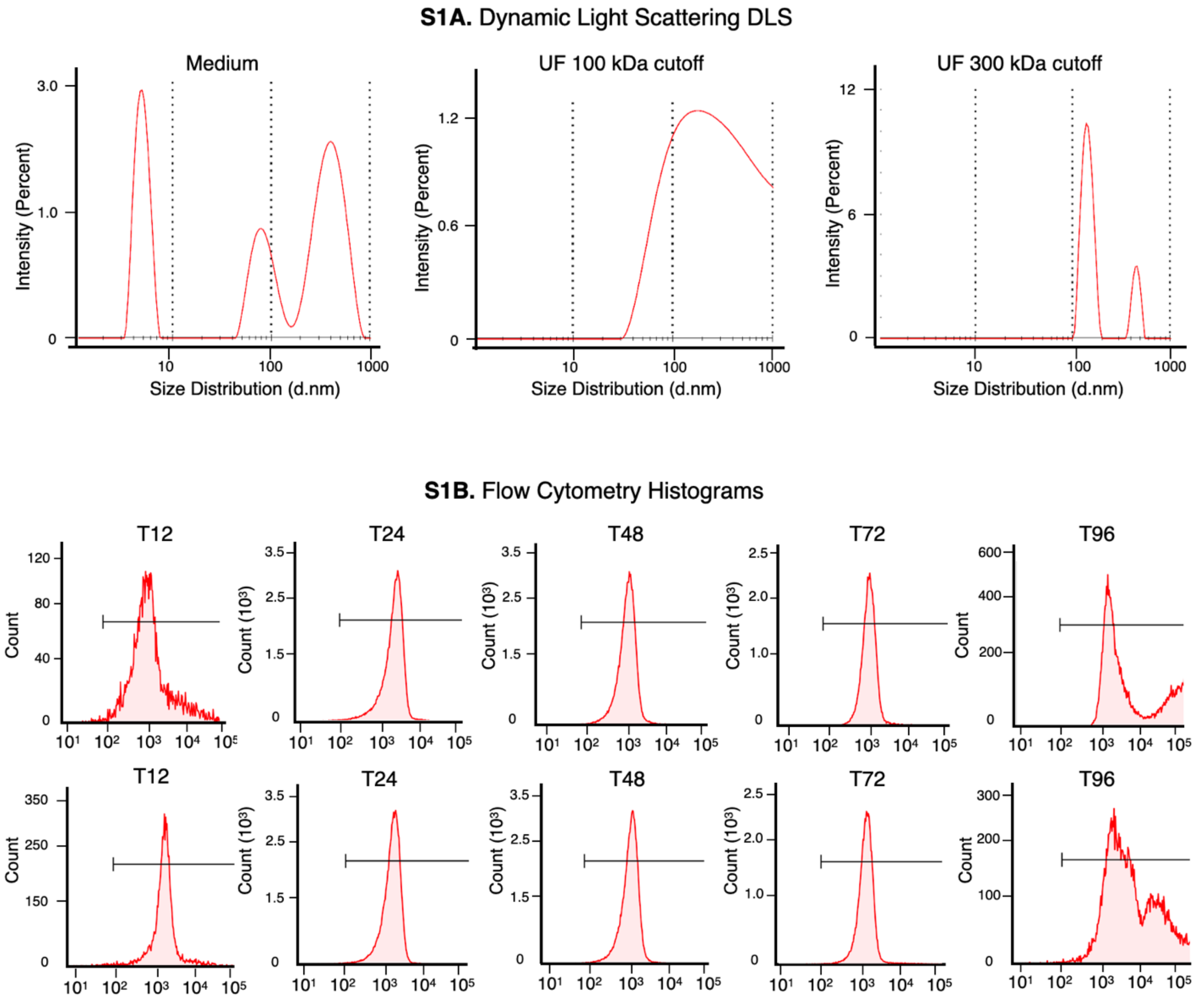

Figure S1 EV detection with Dynamic Light Scattering and Flow Cytometry.

S1A DLS particle size distribution profiles of crude medium and its 100 kDa and 300 kDa ultrafiltrate fractions from *A. nidulans* after 72 hours of cultivation.

Although vesicles smaller than 100 nm are known to occur in fungi, including chitosomes and other cell wall-associated carriers, our analysis indicated that the 300 kDa ultrafiltration cutoff most effectively enriched the secretome-associated extracellular vesicles characterized in this study. Consequently, particles <100 nm may have been missed in the final EV fraction.

S1B FC histograms of CellBrite-stained EVs purified (300 kDa UF) from cultures grown for up to 96 hrs.

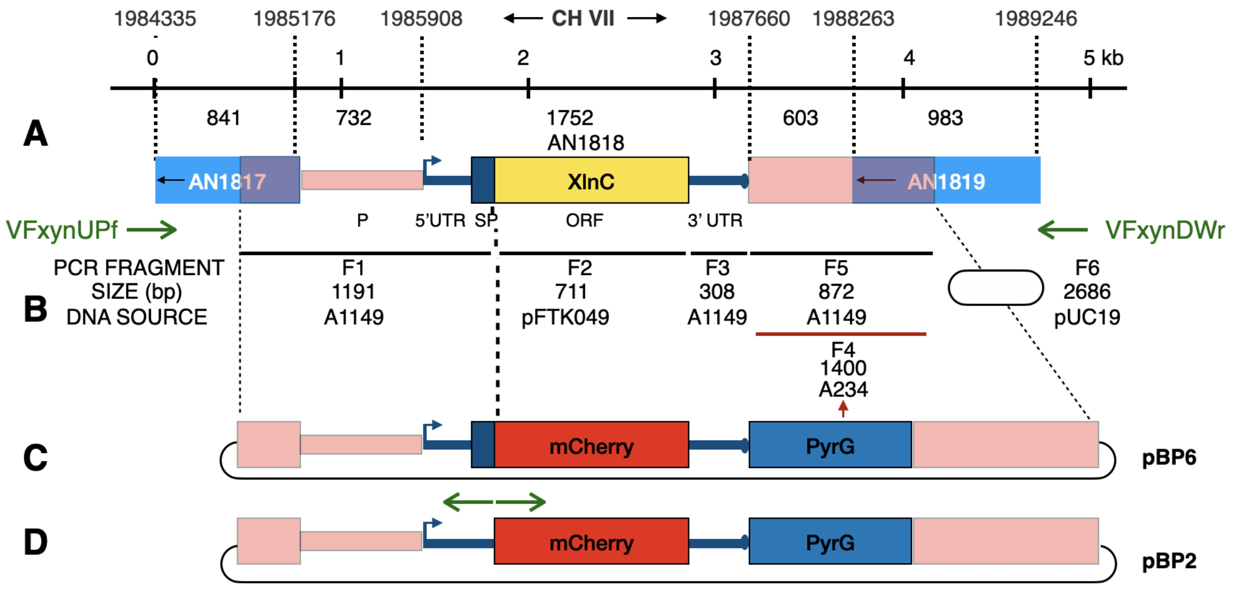

Figure S2 Molecular outline of XlnC replacement with mCherry and signal peptide removal.

S2A illustrates the AN1818 chromosomal region, showing the coordinates and genetic elements subjected to molecular modifications.

S2B depicts the sizes (not to scale) of PCR-amplified DNA fragments (F1-F6) and the corresponding template DNA sources: FGSC A. nidulans strains A1149 and A234, the pFTK049 plasmid containing mCherry (ADDGENE), and the pUC19 vector.

S2C shows the expected genotype of recombinants arising from a double crossover integration at the AN1818 locus.

S2D shows the expected genotype of the strain ΔXynSP, which lacks the signal peptide (SP).

Table S1 - Primers

FG	Size (bp)	Primer Name	Sequence
F1	1191	XCUPf	tcgagctcggtacccggggaatccgaacgatatcggggag
		XCUPr	tcctcgcccttgctcaccatgacaaaaagatcatttaggctgg
F2	711	mCHERRYf	gcctaaatgatctttttgtcatggtgagcaagggcgaggag
		mCHERRYr	caccaaatccaggacaatgcttacttgtataactcgtccatgccg
F3	308	XC3Uf	tggacgagttatacaagtaagcattgtcctggatttggtg
		XC3Ur	gcgttctcgaggaagttgcgtcaagatgctcggccgaactag
F4	1400	PYRGf	agttcggccgagcatcttgacgcaacttcctcgagaacgc
		PYRGr	ccccaagacagagagccgctccccttttagtcaataccgttac
F5	872	XCDWf	acggtattgactaaaaggggagcggctctctgtcttgggg
		XCDWr	tgcaggtcgactctagaggaaatgacaatgacggcggagttg
F6	2686	PUCDWf	actccgccgtcattgtcatttcctctagagtcgacctgcag
		PUCUPr	ctccccgatatcgttcggattccccgggtaccgagctcgaattc
No Sig P construct ΔXynSP		xlnC5UTRf	atccgaacgatatcggggag
		xlnC5UTRr	tcatttaggctggcgctttgtttgggtaagagttgaacgatg
		NSPmCHf	tcgttcaactcttacccaaacaaagcgccagcctaaatgatc
		NSPmCHr	ttacttgtataactcgtccatgc
Genotype validation		VFxynDWr	agcatagaaatggataaaaa
		VFxynUPf	agatcatcgcatgaatgaa

Table S2 Strain used in this work

**Name**	**Genotype**	**Source**
A1149	*pyrG89, pyroA4, ΔkuA::argB*	FGSC
A773	*pyrG89, wA3, pyroA4, ΔkuA::argB*	FGSC
XynMC11	*pyrG89*, *pyroA4*, *ΔkuA::argB*, *ΔxlnC::SP-mCherry*	This work
XlnR7MC7	*paba::gpdA::XlnR::paba, wA3, pyroA4, ΔxlnC::SP-mCherry*	This work
ΔXynSP	*pyrG89*, *pyroA4*, *ΔkuA::argB*, *ΔxlnC::ΔSPmCherry*	This work
